# Casparian strip membrane domain proteins in *Gossypium arboreum*: genome-wide identification and negative regulation of lateral root growth

**DOI:** 10.1186/s12864-020-6723-9

**Published:** 2020-05-04

**Authors:** Xiaoyang Wang, Yuanming Zhang, Liyuan Wang, Zhaoe Pan, Shoupu He, Qiong Gao, Baojun Chen, Wenfang Gong, Xiongming Du

**Affiliations:** 1 State Key Laboratory of Cotton Biology, Institute of Cotton Research, Chinese Academy of Agricultural Sciences, Anyang, 455000 Henan China; 2 Crop Information Center, College of Plant Science and Technology, Huazhong Agricultural University, Wuhan, 430070 China; 3 Key Laboratory of Cultivation and Protection for Non-Wood Forest Trees, Ministry of Education, Central South University of Forestry and Technology, Ministry of Education, Changsha, 410004 China

**Keywords:** Casparian strip membrane domain proteins (*CASPs*), *G. arboreum*, Collinearity analysis, Expression profiles, Lateral root development

## Abstract

**Background:**

Root systems are critical for plant growth and development. The Casparian strip in root systems is involved in stress resistance and maintaining homeostasis. Casparian strip membrane domain proteins (CASPs) are responsible for the formation of Casparian strips.

**Results:**

To investigate the function of CASPs in cotton, we identified and characterized 48, 54, 91 and 94 *CASPs* from *Gossypium arboreum, Gossypium raimondii, Gossypium barbadense* and *Gossypium hirsutum,* respectively, at the genome-wide level. However, only 29 common homologous *CASP* genes were detected in the four *Gossypium* species. A collinearity analysis revealed that whole genome duplication (WGD) was the primary reason for the expansion of the genes of the *CASP* family in the four cotton species. However, dispersed duplication could also contribute to the expansion of the *GaCASPs* gene family in the ancestors of *G. arboreum*. Phylogenetic analysis was used to cluster a total of 85 *CASP* genes from *G. arboreum* and *Arabidopsis* into six distinct groups, while the genetic structure and motifs of *CASPs* were conserved in the same group. Most *GaCASPs* were expressed in diverse tissues, with the exception of that five *GaCASP*s (*Ga08G0113, Ga08G0114*, *Ga08G0116*, *Ga08G0117* and *Ga08G0118*) that were highly expressed in root tissues. Analyses of the tissue and subcellular localization suggested that *GaCASP27* genes (*Ga08G0117*) are membrane protein genes located in the root. In the *GaCASP27* silenced plants and the *Arabidopsis* mutants, the lateral root number significantly increased. Furthermore, *GaMYB36*, which is related to root development was found to regulate lateral root growth by targeting *GaCASP27.*

**Conclusions:**

This study provides a fundamental understanding of the *CASP* gene family in cotton and demonstrates the regulatory role of *GaCASP27* on lateral root growth and development.

## Background

Many higher plants have enormous and complex root systems, which are regulated by lateral roots [1]. Lateral roots are hidden below the ground, and play an important role in providing nutrients and water, which supports the rest of the plant. A large root system supports plant growth and development, and ultimately increases crop yield. For some dicotyledonous plants such as cotton, the lateral roots grow from the primary roots [2, 3].

Plant roots absorb nutrients from the soil through the symplast or apoplastic pathways. However, when the nutrients and water reach the root endodermis, the apoplastic pathway is blocked. To keep moving nutrients to the aerial parts, it must rely on plasma membrane special carrier proteins [4]. Blocking apoplastic flow allows the plants to adapt to various environmental changes [4]. This specialized structure forms a hydrophobic band called the “Casparian strip” [5]. When defective mutants are present in the Casparian strip, it fails to maintain ion homeostasis due to the inward or outward leakage of xylem ions, which causes abnormal phenotypes in adverse soil conditions [6]. The formation of the Casparian strips depends on Casparian strip membrane domain proteins (CASPs), which are primarily responsible for the accumulation of lignin polymerization in the central region of endodermal plasma membranes [7]. Five Casparian strip membrane domain proteins, containing four transmembrane domains were identified in *Arabidopsis* roots, CASP1 and CASP3 play vital roles during the formation of Casparian strips. The CASPs are located in the plasma membrane, and interact with secreted peroxidases, directly modifying the cell wall with their membrane domain [8, 9]. Furthermore, the peroxidase (*PRX*) genes and monolignol oxidizing enzyme genes, such as *Respiratory burst oxidase homologue F* (*RBOHF*) and *laccases* (*LACs*) are preferentially accumulated in the endodermis, which are necessary for the formation of Casparian strips [10]. The plant peptide hormone, named “Casparian strip integrity factor” (CIF1/2), binds to the leucine-rich repeat receptor kinase SCHENGEN3 (SGN3) and is necessary for the contiguous formation of Casparian strips [6]. The *SGN3* mutants severely disrupted the Casparian strips without altering the concentration of most ions, with the exception of magnesium and potassium homeostasis [11]. SGN1, a receptor-like cytoplasmic kinase (RLCK), ensures the Casparian strip membrane domain is in the correct position [12]. The transcription factor *SHORTROOT* (*SHR*) targets another transcription factor, MYB36, and is also responsible for the development of Casparian strips [13].

Cotton is a natural fiber from which textiles are manufactured, and also provides cottonseed oil [14]. Cotton root systems include one primary root and numerous lateral roots, while the lateral root development affects the whole root system. Larger cotton root systems can resist nutrient deficiency by efficiently absorbing K^+^ [15, 16]. However, the restricted root system reduces cotton photosynthesis and biomass production [17]. Some genes play important roles in regulating cotton lateral root development: *GhARG*, a cotton arginase gene, represses the formation of the lateral root. The *GhARG* silenced cotton plants that were grow well under both low and high nitric conditions [18, 19]. Additionally, the *GhSTOP1* gene positively affects lateral root development when exposed to acid stress [20]. Using RNAi technology to down-regulate *GhSTOP1* in cotton decreases the expression of genes related to lateral root development and delays the growth of lateral roots.

Compared with allotetraploid cotton which has a complex genome (AADD), diploid Asiatic cotton (*G. arboreum*) has a simple genome (AA), which makes it a valuable resource for studying agricultural and morphological traits. The published genomic data of *G. hirsutum*, *G. barbadense*, *G. raimondii* and *G. arboreum* helps analyze of *CASP* genes at the genome-wide level in cotton. However, the specific function of the CASP gene family in *Gossypium* is largely unknown. The relationship between CASP proteins and root development in cotton has yet to be identified. In this study, 48 *CASP* genes were identified. They were randomly distributed on all 13 chromosomes of *G. arboreum*. Among them, 29 were identified in the four *Gossypium* species. Most *CASP* genes exhibited a high level of expression in the initial growth stages of fiber, and roots, stems and leaves in vegetable tissues. However, five *GaCASP* genes (*Ga08G0113*, *Ga08G0114*, *Ga08G0116*, *Ga08G0117* and *Ga08G0118*) were exclusively expressed in roots. Histochemical analysis showed that *GaCASP27* was particularly expressed in roots. This study outlines findings that will aid in the further identification of the functions of *CASP* genes in root development, which can be utilized to breed new varieties with large root systems.

## Results

### Identification and chromosomal distribution analysis of *CASP* genes in *Gossypium*

Our previous study identified four Casparian-strip membrane protein genes in 215 *G. arboreum* accessions [21]. These homologous genes included *Ga08G0114*, *Ga08G0116*, *Ga08G0117*, and *Ga08G0118*. This study used *Ga08G0117* sequence as a query, and downloaded PF04535 from Pfam database (http://pfam.xfam.org/) using Hidden Markov Model (HMM) analysis [22]. We searched the cotton protein dataset using the HMMER3.0 software [23]. A total of 49, 57, 110, and 101 CASP genes were identified from *G. arboreum* (diploid), *G. raimondii* (diploid), *G. barbadense* (tetraploid), and *G. hirsutum* (tetraploid), respectively. The putative CASP genes were then analyzed using SMART (Simple Modular Architecture Research Tool) and NCBI-CDD databases (https://www.ncbi.nlm.nih.gov/cdd) to identify the common domain of the CASPs, using the threshold (E < 10^− 14^). Finally, 48, 54, 91 and 94 CASP genes were identified from *G. arboreum, G. raimondii, G. barbadense* and *G. hirsutum,* respectively. The genes *GaCASP1* to *GaCASP48*, *GbCASP1* to *GbCASP91*, *GrCASP1* to *GbCASP54* and *GhCASP1* to *GhCASP94* were named based on their genetic IDs in the genome database. The chromosomal locations of the CASPs were collected from cotton FGD (https://cottonfgd.org/) and are listed in Additional file 2. These genes were unevenly distributed on different *Gossypium* chromosomes. The chromosomal distribution of *GaCASP* genes was constructed by the Mapchart software based on their chromosomal location (Additional file 3, Additional file 8). *GaCASP* genes were randomly distributed over all 13 chromosomes of *G. arboreum.* The *Ga08G0114*, *Ga08G0116*, *Ga08G0117*, and *Ga08G0118* genes were located on the same Chr8, indicating that these genes could have been duplicated from an identical gene and perform similar functions. However, the *CASP* gene *Ga14G0035* was not mapped on any chromosomes (Additional file 8). Furthermore, consecutively numbered genes in the same chromosome showed similar molecular weights and lengths of proteins, such as the diploid Asiatic cotton genes *Ga08G0113* and *Ga08G0114*, *Ga08G0116*, *Ga08G0117* and *Ga08G0118*, the tetraploid upland cotton genes *Gh_A08G0061*, *Gh_A08G0062*, *Gh_A08G0063*, *Gh_A08G0064* and *Gh_A08G0065*, the tetraploid island cotton genes *GOBAR_DD14175*, *GOBAR_DD14176*, *GOBAR_DD14177*, and *GOBAR_DD14178*, and the diploid D genome genes *Gorai.004G010700.1*, *Gorai.004G010800.1*, *Gorai.004G010900.1*, *Gorai.004G011000.1*, and *Gorai.004G011100.1*. Among all of the identified CASP proteins, GhSca109203G01 was the smallest protein, with 69 amino acids (aa). The largest was Gh_D09G1628 (325 aa). The molecular weight of the proteins ranged from 7.746 to 35.878 kDa, while the isoelectric point ranged from 3.894 (Gh_A10G1948) to 11.672(Gorai.012G052700.1). Detailed information regarding Casparian strip proteins in cotton is available in Additional file 2.

### Collinearity analysis of 48 Casparian strip genes from *G. arboreum* compared with *G. barbadense*, *G. hirsutum* and G. raimondii

**Fig. 1:**
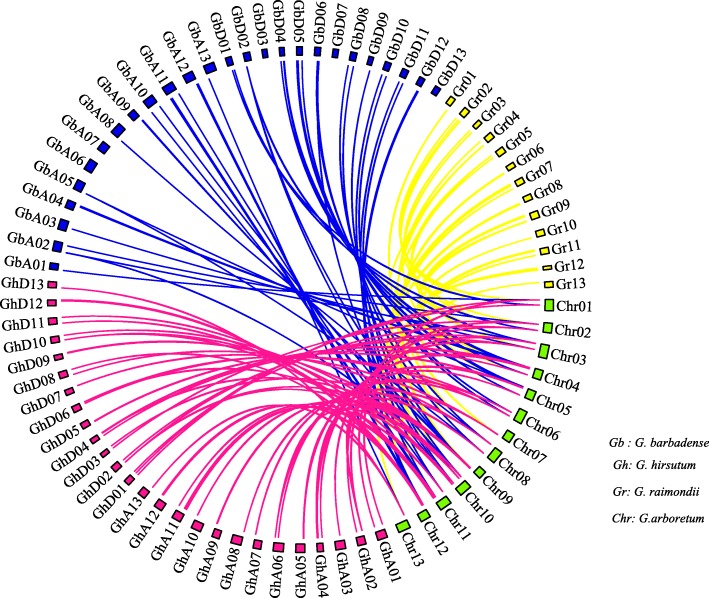
Microsynteny analysis of *CASP genes* between *G. arboretum and G. hirsutum, G. barbadense* and *G. raimondii*. Red lines connect the homologous genes between *G. arboretum* and *G. hirsutum*, yellow lines connect the homologous genes between *G. arboretum* and *G*. *raimondii*, and blue lines connect the homologous genes between *G. arboretum* and *G. barbadense*. Green, pink, blue, and yellow boxes indicate the *G. arboretum*, *G. hirsutum*, *G. barbadense* and *G*. *raimondii* chromosomes, respectively

Previous studies have demonstrated that whole-genome, tandem and segmental duplications play central roles in the expansion of the *Gossypium* gene family [24, 25]. A chromosomal region within 200 kb containing two or more consecutive genes is defined as a tandem duplication event [26]. To reveal the genome-wide duplicated mechanism of the CASP gene family in *G. arboreum*, all intragenomic duplication data was filtered by MCScanX. Four tandem duplicated gene pairs (*Ga03G0527.1/Ga03G0528.1*, *Ga08G0113.1/Ga08G0114.1*, *Ga08G0116.1/Ga08G0117.1*, and *Ga08G0116.1/Ga08G0117.1*) were detected in *G. arboreum.* Nine tandem duplicated gene pairs were detected in *G. hirsutum* and *G. barbadense*, while no tandem duplicated gene pairs were detected in *G. raimondii*. Throughout the whole genomic analysis, 18, 44, 66 and 72 gene pairs were considered whole genome duplication (WGD) in *G. arboreum*, *G. raimondii*, *G. barbadense* and *G. hirsutum*, respectively. There are 21, 10, 10 and 10 gene pairs that are considered dispersed duplications in *G. arboreum*, *G. raimondii*, *G. barbadense* and *G. hirsutum*, respectively. Two gene pairs were detected as proximal duplications in *G. arboreum*. However, other *gossypium* did not detect proximal duplication of the CASP gene family. As a result, whole genome and dispersed duplications could be the primary driving forces for the expansion of the *CASP* gene family in *Gossypium* (Additional file 4). Tandem duplication events occurred less frequently, suggesting they it might not play a key role in the expansion of *GaCASPs, GrCASPs, GbCASPs and GhCASPs*. A total of 70, 45 and 39 orthologous CASP gene pairs were detected between *G. arboreum* and *G. hirsutum*, *G. barbadense* and *G. raimondii* using TBtools software, respectively. We identified a total of 29 common homologous *CASP* gene pairs in the four *Gossypium* species. Details for the collinear gene pairs are listed in Fig. 1 and Additional file 5.

### Phylogenetic analysis and classification of CASP genes

**Fig. 2:**
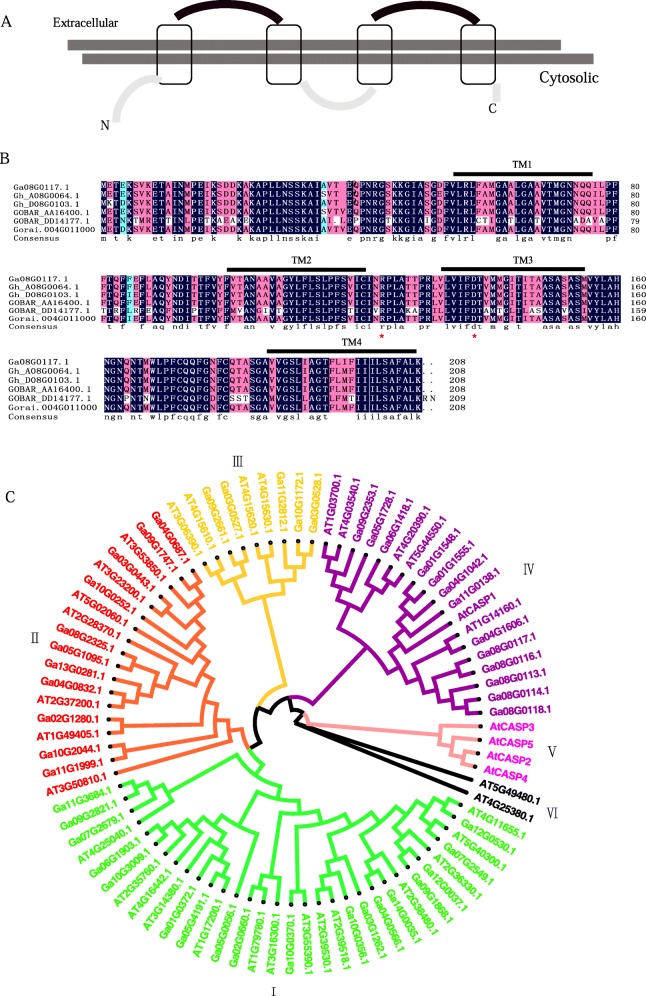
Topology, conserved Casparian strip membrane domain features, and phylogenetic tree of the CASP family. **a** Predicted topology of the GaCASPs, the four gray boxes represent the four-membrane spans, the arc lines indicate cytosolic amino, carboxy termini, and conserved extracellular loops. **b** Multiple alignment and transmembrane region analysis of GaCASP, GhCASP, GrCASP, and GbCASP protein sequences. The four transmembrane (TM) domains were analyzed using the TMHMM program. **c** Phylogenetic relationships of CASPs between *Arabidopsis* and *G. arboretum*. The phylogenetic tree was constructed by the MEGA 7 program based on the protein sequences. The maximum likelihood method was used and bootstrap values were carried out 1000 replications

Members of the GaCASPs family have conserved extracellular loops as well as the standard topology of four-membrane spans with cytosolic amino and carboxy termini (Fig. 2a), which is consistent with those found in *Arabidopsis* [7]. Furthermore, we randomly selected six CASP genes (*Ga08G0117, Gh_A08G0064, Gh_D08G0103, GOBAR_AA16400.1, GOBAR_DD14177.1 and Gorai.004G011000*) from each clade to perform further protein sequence analyses. The CASP paralogs proteins were highly conserved in amino acids, which contained a domain that could have catalytic activity with a conserved arginine and aspartate, forming an active site (Fig. 2b). These proteins contain four transmembrane helices. In order to understand the similarities and differences in GaCASPs between cotton and *Arabidopsis*, the phylogenetic tree was constructed using 48 CASP protein sequences from *G. arboreum* and 37 CASP sequences from *Arabidopsis*. Subsequent phylogenetic analysis indicated that CASPs were mainly grouped into six separate subfamilies (Fig. 2c). Clade I had 32 members, followed by Clade IV (19), CladeII (18) and CladeIII (9). Clade V had only two members, while Clade V and VI contained only *Arabidopsis* genes.

### Gene structure and conserved motif composition of *G. arboreum* CASP gene family

**Fig. 3:**
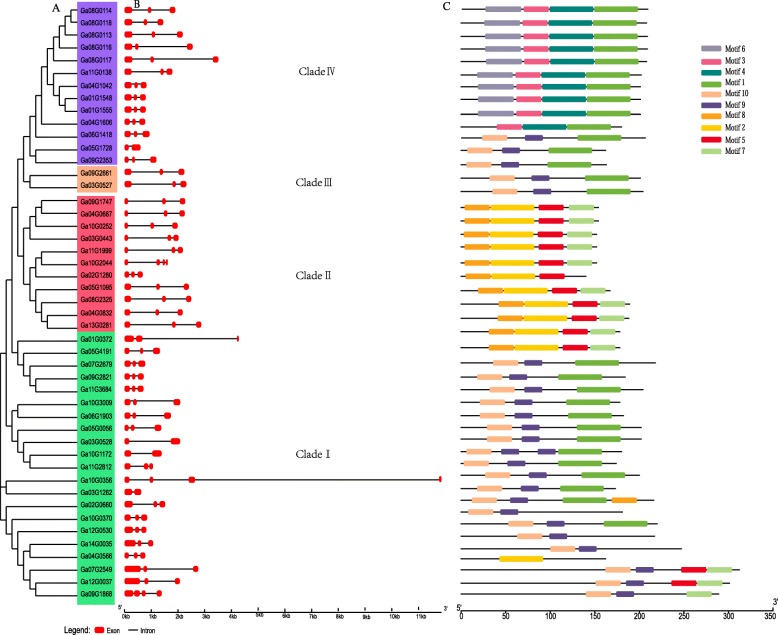
Phylogenetic tree, exon-intron structures and motif composition of *CASP* genes in *G. arboretum*. **a** The phylogenetic tree was constructed using the MEGA 7 program. **b** Schematic diagram for the exon/intron organization of *GaCASPs*. The red boxes and black lines represent the exons and introns, respectively. **c** The conserved protein motifs in the *GaCASPs* were identified using MEME online software. Each motif is associated with a specific color

To gain more insight into the evolution of the *GaCASP* family in *G. arboreum*, we examined how the exons and introns were organized in all the identified *GaCASP* genes and constructed a phylogenetic tree using 48 GaCASPs protein sequences. Forty-eight genes were divided into four groups, while most *CASP* genes typically contained three exons and two introns (Fig. 3a). *Ga08G0113*, *Ga08G0114*, *Ga08G0116,* and *Ga08G0118* possessed a similar exon-intron structure, but the *Ga08G0117* gene possessed a long exon-intron structure (Fig. 3b). Further analysis of the MEME motif was used to predict the protein-conserved motifs in GaCASPs. Ten distinct motifs were identified, while GaCASP proteins in the same group typically shared a similar motif composition (Fig. 3c). Nine motifs were identified in Clade I, with the exception of motif 4. Clade II contained motif 8, motif 2, motif 5,and motif 7. Clade III contained motif 1, motif 9, and motif 10. Clade IV contained motif 6, motif 3, motif 4, and motif 1. Overall, the GaCASP members of the same group shared similar conserved motif compositions and gene structures. Along with the results of our phylogenetic analysis, this strongly supports the reliability of the group classification results.

### Expression profiling of *G. arboreum* CASP genes with RNA-seq

**Fig. 4:**
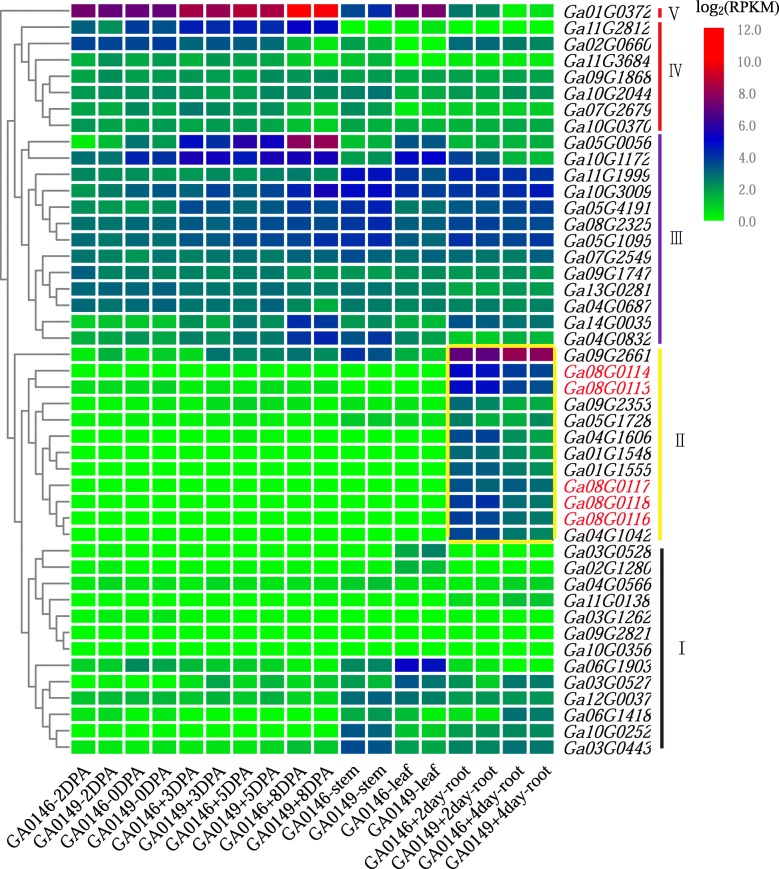
Transcript analysis of the CASP family genes in the different tissues of *G. arboretum* varieties. Expression levels were shown as log_2_(RPKM). The heat map was constructed using TBtools based on the expression data. The hierarchical clusters were generated according to the characteristics of *CASP* genes expression in different tissues. The yellow box displays genes specifically enriched in roots. The green colors indicate low expression levels, while red represents high expression levels. The gene expression data of each sample has three replicates, with three samples collected for each

In order to elucidate the possible role of CASPs in the root growth and development of *G. arboreum*, we investigated the expression patterns of 48 *GaCASPs* in different developmental stages of fiber, root, stem and leaf tissues using the transcriptome data, (Additional file 6 and Fig. 4). In order to further classify the gene expression patterns of the 48 *GaCASPs* gene, these genes were classified using hierarchical Clustering Software Cluster3.0 following their statistical analysis. The expression patterns were then divided into five major clusters, based on tree branching. Most *CASP* genes were expressed in all tissues, but some *CASP* family members were only expressed in the roots, such as the genes in group II. Only one gene, *Ga01G0372* (classified into the fifth group) was significantly expressed in different stages of fiber development but showed weaker expression in roots. The genes in the first group displayed low expression levels in all of the detected tissue. The genes in the second group were highly expressed in roots. As shown in the yellow box, these genes included: *Ga08G0113*, *Ga08G0114*, *Ga08G0116*, *Ga08G0117,* and *Ga08G0118* (red letters) as well as other *CASP* like genes in this family. The third group contained 13 genes that were highly expressed in all the detected tissues. The gene *Ga11G2812* (in the fourth group) was expressed across the different development stages of fibers, however, no expression was detected in the vegetable tissues (stems, leaves and roots). These results indicated that certain *CASPs* had different spatial and temporal expression patterns in cotton.

### Tissue expression and subcellular localization of GaCASP3 protein

**Fig. 5:**
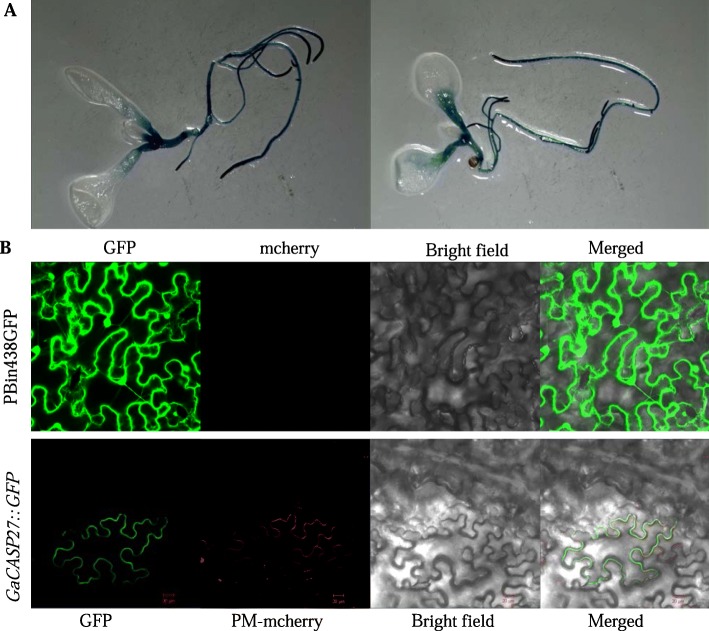
The tissue and subcellular localization of *GaCASP27.* **a** GUS-staining analysis of the p*GaCASP27::*GUS transgenic *Arabidopsis* in primary roots. **b** Subcellular localization of *GaCASP27* in *N. benthamiana.* GaCASP27::GFP signal was merged with that of the PM-mCherry (an intrinsic plasma membrane protein) marker in *N. benthamiana.* The vector of *pBinGFP438* was used as the control*.* All experiments were analyzed with three biological repeats

In order to further confirm the localization of expression of *CASP* genes, we selected the *GaCASP27* gene (*Ga08G0117*) to construct a *GaCASP27*-promoter-GUS (for β*-*glucuronidase) reporter vector, followed by transformation into *Arabidopsis* (Additional file 9). We observed intense GUS staining in the roots (Fig. 5a), indicating that *GaCASP27* was primarily expressed in roots.

According to the online tools TargetP and SignalP, the GaCASP27 protein should be localized in the plasma membrane. Subcellular localization of the GaCASP27 protein was determined by the construction of a GaCASP27-green fluorescent protein (GFP) fusion under the control of *CaMV35S* promoter. The plasma membrane markers red fluorescent protein (RFP) and *35S-GaCASP27::GFP* were co-transformed into the leaves of tobacco (*Nicotiana benthamiana*). Laser confocal microscopic results revealed that the empty control vector pBin438GFP presented a fluorescent signal in the cell membrane, nucleus and cytoplasm. By contrast, *GaCASP27::GFP* fusion proteins were co-localized with a plasma membrane marker in the plasma membrane, which confirmed our hypothesis (Fig. 5b). These results indicated that the GaCASP27 protein is a membrane protein.

### Silencing *GaCASP27* increased the lateral root number

**Fig. 6:**
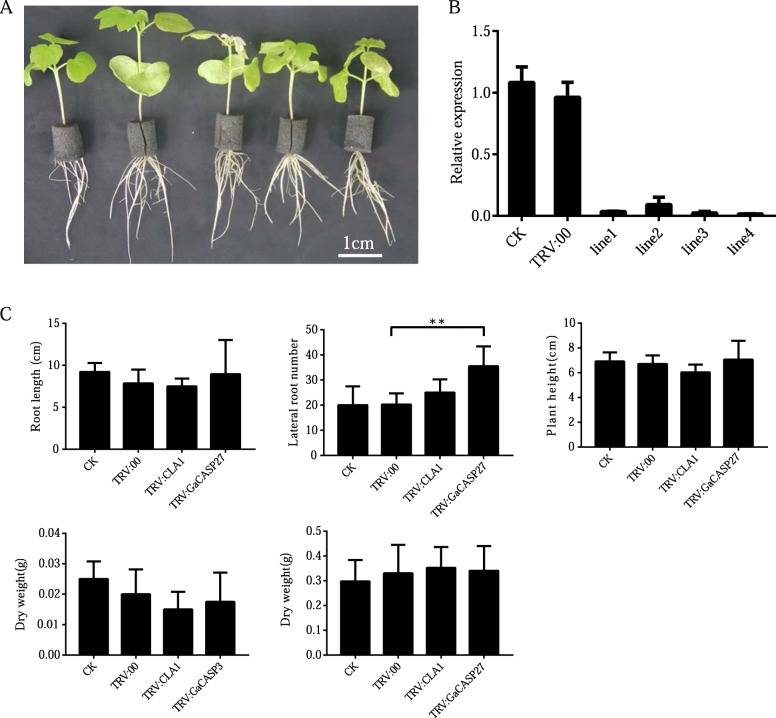
*GaCASP27* silenced plants have an increased number of lateral root phenotypes in *G. arboreum*. **a** Phenotypes of gene silencing plants were observed 2 weeks after infiltration. The plants from left to right were blank control (CK), negative control (TRV:00), positive control (TRV: CLA1) and two *GaCASP27*-silenced lines. **b** The expression levels of *GaCASP27* in the blank control, negative control and silenced cotton plants were conducted through qRT-PCR All experiments were analyzed with three independent biological replicates. **c** Two weeks after infiltration, the root length, lateral root number and plant height were determined in GA0149 accession. All experiments were analyzed based on three independent biological replicates. The significant difference analysis was performed using the t-test at *P* ≤ 0.05

The virus-induced gene-silencing (VIGS) method was used to further validate the functional role of *GaCASP27* in root development. A 311 bp fragment in the 3′ end of *Ga08G0117* was isolated and cloned into pTRV1 to construct the TRV:GaCASP27 vector [27]. The empty vector TRV:00 was used as a negative control. After 2 weeks of *Agrobacterium*-based infiltration, the cotton plants harboring TRV:CLA1 showed photobleaching phenotype in young leaves, indicating that the VIGS experiment was successful (Fig. 6a). To further determine the gene silencing efficiency, qRT-PCR was used to evaluate the expression levels of *GaCASP27* in both the TRV:GaCASP27 and TRV:00 control cotton plants. Compared with TRV:00 control plants, the expression level of *GaCASP27* was drastically reduced in the TRV:GaCASP27 plants (Fig. 6b). The number of lateral roots in *GaCASP27*-silenced plants increased compared with the bank (CK) and negative control plants (Fig. 6c). The *CASP* genes *Arabidopsis* mutants *Atcasp1/2/3* also exhibited a greater number of lateral roots compared with WT (Additional file 10). These findings suggest that the Casparian strip gene *GaCASP27* negatively regulates the development of lateral roots in cotton and *Arabidopsis*.

### *GaCASP27* was a target gene of *GaMYB36*

**Fig. 7:**
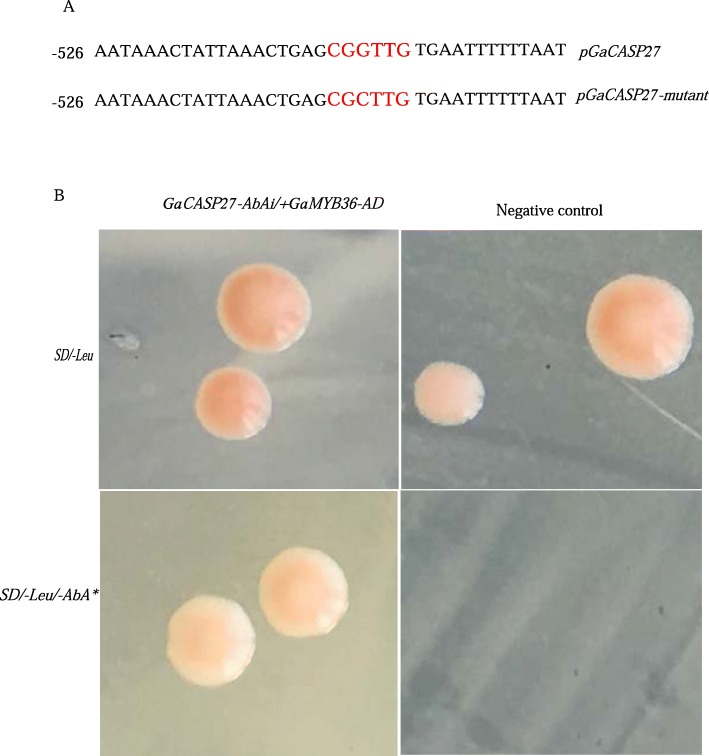
*GaMYB36* binds to the MBSI sequences of the *GaCASP27* promoter in yeast. **a** The 2.0 kb *GaCASP27* promoter region upstream of the ATG start codon includes predicted MBSI sequences (CGGTTG). **b** A yeast one-hybrid assay was performed to analyze the interaction between *GaMYB36* and *GaCASP27*. Three biological repeats were performed

Previous studies demonstrated that *AtMYB36* could bind to the promoter of *AtCASP* via chromatin immunoprecipitation (ChIP)-qPCR [13]. The point mutation *Atmyb36–1* had longer primary roots and misshaped lateral roots in early stages [28, 29]. Our results also showed that *Atmyb36–1* had null lateral roots (Additional file 10). In order to characterize the genetic interactions between *MYB36* and *GaCASP27*, we analyzed the promoter element of *GaCASP27* and identified seven MYB *cis*-elements MBSI CNGTT(A/G) (Fig. 7a and Additional file 7). We then performed a yeast one-hybrid assay (Y1H). *GaMYB36* was bound to the MBSI sequence of the *GaCASP27* promoter and grew on SD medium containing the Leu/AbA (Fig. 7b). These results indicate that *GaMYB36* plays an important role in regulating lateral root development by targeting *GaCASP27.*

## Discussion

### Characterization of Casparian gene family

Casparian strips are present in the roots of euphyllophytes, such as ferns and spermatophytes. They are involved in the defense against various biotic and abiotic stresses [28, 30–33]. Casparian strips also function as a receiver, hormone transporter, signal of calcium waves, respond to external environmental changes, and promote the growth and development of new lateral organs. Proteins in the CASP family recruit a group of enzymes catalyzing lignin biosynthesis [34, 35]. However, the roles of *CASP* genes in the development of cotton roots have yet to be well studied.

In this study, we isolated 48, 91, 94 and 54 *CASP* genes from *G. arboreum*, *G. barbadense*, *G. hirsutum* and *G. raimondii*, respectively. Interestingly, the number of *CASP* genes in *G. hirsutum* was almost equal to that of *G. raimondii* and *G. arboreum*. This could be because the allotetraploid cotton genomes are composed of diploid A and D genome [36]. The number of *CASP* genes in cotton was more than that of *Arabidopsis*. The likely reason that cotton higher has more *CASP* genes is that it has a more complex genome. Tandem, whole-genome, and segmental duplications are the three major mechanisms contributing to the complexity of the genomic structure in higher plants over evolutionary time [37]. Gene duplicating is often related to forming of homologous genes in gene families [38]. Tandem duplication is usually caused by unequal crossings, while many of these occurrences can lead to the expansion or reduction of different gene family members [39]. Wang (2019) demonstrated that tandem replication within *At* and *Dt* sub-genomes causes the expansion of the *GhSOT* gene family [40]. The WGD known as polyploidization, which results in double chromosomes and retained duplicates, is considered a primary driver of species diversification [41]. Wang (2018) found that WGD was the determining factor for the expansion of the *GSK* gene family [42]. Proximal duplication includes two gene copies that are closely located on the chromosome, but separated by a few genes [43]. Two adjacent gene duplications that originated from ancient tandem duplication events can be destroyed by inserting other genes, which is the source of proximal duplication [44]. Furthermore, the activity of localized transposon can lead to proximal duplication [45]. After performing a collinearity analysis, we identified the gene duplication events among *CASP* genes, and detected four tandem, eighteen whole-genome and two proximal duplication gene pairs in *G. arboreum* (Additional file 4). Dispersed duplicates create two gene copies that are neither neighboring to each other in the genome nor within paralogous chromosome segments [46]. Dispersed duplicates include transposable elements that are ubiquitous in the human genome, such as members of the *Alu* and *L1* family [47]. Stewart (2011) detected 1000 dispersed duplications using a database-free approach in the human genome [48]. The genome-wide analysis showed that 21 *GaCASP* genes were produced by dispersed duplication. This suggests that dispersed duplication could have contributed to the expansion of the *GaCASP* gene family in diploid *G. arboreum* ancestors.

Orthologs genes could share identical biological functions over evolutionary time [49]. In this study, 70, 39, and 45 orthologous *CASP* gene pairs were predicted between *G. arboreum* and *G. hirsutum*, *G. arboreum* and *G. raimondii*, and *G. arboreum* and *G. barbadense*, respectively (Additional file 5). CASPs from *G. arboreum* and *Arabidopsis* were divided into six subfamilies using a phylogenetic tree analysis. We observed that in the fourth group, five *CASP* genes *(Ga08G0113, Ga08G0114, Ga08G0116, Ga08G0117,* and *Ga08G0118*) shared close relationships with *Arabidopsis AtCASP1*, which regulated the development of lateral roots. Additionally, we aligned the protein sequences of GbCASPs*,* GaCASPs*,* GrCASPs and GhCASPs*,* and found that all of these CASPs contained four conserved transmembrane domains and two traces of active amino acids, suggesting that these proteins were highly conserved across species.

Gene structure determines gene function [50]. Both motif domain and analysis of gene structure demonstrated that *CASP* genes in the same subgroup shared a similar structure, suggesting that these *CASP* genes may have similar functions. The transcript analysis of *GaCASP* genes revealed that the same group of genes shared similar expression patterns in cotton. Previously published transcriptomic results revealed that *CASP-like* family genes are expressed in various tissues, with similar expression trends in the same group observed in *Arabidopsis* [51]. However, a higher root expression profile was reported for many genes in this family, including other *CASP-like* genes as well as *CASP1/2/3/4/5* [51]. Our study identified five *GaCASP* genes, such as *GaCASP24/25/26/27/28*, that were specifically expressed in roots. This phenomenon prompted us to hypothesize that there is a relationship between *GaCASP* genes and root development.

### *GaMYB36* involvement in root development by interacting directly with lateral root negative regulator *GaCASP27*

*MYB36* plays a critical role in the formation of Casparian strips by the localization of lignin polymerization in *Arabidopsis*. *MYB36* positively regulates cells, in *Arabidopsis* roots, from proliferation to differentiation [28]. The length of the primary root was significantly shorter in lines with *MYB36* overexpression. However, the number of meristem cells increased in lines with the *myb36* mutant [28]. In our study, we observed longer roots and less lateral root density in the *Atmyb36* mutant plants, which is consistent with previous results (Additional file 10). According to Li (2018), MYB36 combines with the transcription factors SCARECROW (SCR) and SHORTROOT (SHR) to form a complex (SHR-SCR-MYB36), regulating the expression of *CASPs* and affecting Casparian strip formation [52]. However, in our study, *MYB36* was directly bound to the promoter of *GaCASP27* and positively regulated the expression of *GaCASP27*. Casparian strip formation plays a critical role in maintaining ROS homeostasis which negatively regulated lateral root development in *Arabidopsis* [53]. This was consistent with our study: the number of lateral roots increased in the *GaCASP27* silenced plants. Furthermore, the single mutants *Atcasp1*, *Atcasp2* and *Atcasp3* possessed more lateral roots compared with WT (Additional file 10). This suggests that *MYB36* is involved in root development by directly interacting with *GaCASP27* (which was mainly responsible for the formation of the Casparian strip) and negatively regulates the growth and development of lateral roots.

## Conclusions

We identified 48, 54, 91, and 94 CASPs from *G. arboreum*, *G. raimondii*, *G. barbadense*, and *G. hirsutum* at the genome-wide level, respectively. Only 29 homologous *GaCASPs* gene pairs were detected in the four *Gossypium* species. WGD might be the primary reason for the expansion of CASPs in these four cotton species. Details about the on the phylogeny, gene structure, conserved motif composition, and physical positions of the chromosomes illustrated both the evolution and conservation of *CASP* genes. Most *GaCASP* genes were expressed across all of the different development stage of fiber, and root, stem and leaf tissues. However, five *GaCASP24/25/26/27/28* genes were highly expressed in roots. Histochemical analysis and subcellular localization further demonstrated that the membrane protein GaCASP27 was also expressed in the roots. Additionally, a VIGS mediated knockdown analysis showed the *GaCASP27* negatively regulated the development of lateral roots in cotton.

## Methods

### Identification and chromosomal distribution analysis of *GaCASPs* in *Gossypium*

The data of *G. raimondii* (JGI), *G. arboreum* (CRI), *G. hirsutum* (NAU) and *G.barbadense* (HAU) were downloaded from the CottonFGD (https://cottonfgd.org/about/download.html), while the data of the protein sequence of *Arabidopsis* were downloaded from TAIR (https://www.arabidopsis.org/). The hidden Markov model (HMM) seed file (Stockholm format) of the uncharacterized protein domain (PF04535) was downloaded from the Pfam website (http://pfam.xfam.org/) and used as a query when searching for Casparian strips (CASP) sequences using the HMMER software (http://hmmer.org). The conserved, uncharacterized protein domains were confirmed by the SMART (http://smart.emblheidelberg.de/), Pfam (http://pfam.xfam.org/), and NCBI-CDD databases. The biophysical properties of the CASP proteins were obtained from CottonFGD (https://cottonfgd.org/about/download.html). Information about the chromosomal position of the *GaCASPs* genes was obtained from CottonFGD (https://cottonfgd.org/analyze/), while the “*GaCASPs* distribution map” was drawn by MapChart [54].

### Gene collinearity and duplication analysis

The TBtools software (https://github.com/CJ-Chen/TBtools) was used to analyze collinearity pairs of *CASP* genes from *G. arboreum* and three other *Gossypium* species. MCScanX (http://chibba.pgml.uga.edu/mcscan2/) was used to analyze gene duplication events in *G. arboreum* genomes [55]. Tandem duplication events were identified when two consecutive genes were distributed on the same chromosomes under the threshold of e < 10^− 20^. Segmental duplications were defined as duplication events within large chromosomal regions in the same chromosom [37, 56]. Segmentally duplicated pairs among CASP proteins from *G. arboreum* and the three other *Gossypium* accessions were analyzed using TBtools (https://github.com/CJ-Chen/TBtools). Tandem duplications were identified using a BLAST-Like Alignment Tool (BLAT) [57].

### Characterization and sequence analysis of related *CASP* genes

The genomic and coding sequences of the *CASP* genes were downloaded from TAIR (https://www.arabidopsis.org/) and cottonFGD (https://cottonfgd.org/) and analyzed using the Gene Structure Display Server online software (GSDS2.0) (http://gsds.cbi.pku.edu.cn/) to predict gene structures. Multi-protein sequence alignment of the CASPs was conducted by the DNAMAN software, while the phylogenetic tree was built using the MEGA 7 software with the maximum likelihood (ML) and JTT + G models [58–61]. We performed 1000 bootstrap replications to produce bootstrap values. The identification of the transmembrane region for *GaCASPs* was predicted by the TMHMM program [51]. The protein sequences of GaCASPs were submitted to the MEME online software (http://meme-suite.org/tools/meme) to predict the conserved motif.

### Plant materials and seedling growth

The varieties of diploid Asiatic cotton (*G. arboreum* L.) wildtype DPL971(GA0146) and fuzzless mutant DPL972 (GA0149) were introduced from Yunnan province, China, before 1998 and kept in the Chinese National Germplasm Mid-term Genebank (Anyang, China) [62]. In our present study, these two varieties were provided by Mid-term Genebank. The roots of GA0149 and GA0146 were harvested from seedlings at two- and four-old-day development stages, while the fresh leaves and stems were harvested from three-week-old seedlings grown in the green house. Samples of fiber and ovules were collected in the morning at − 2, 0, + 3, + 5 and + 8 day post anthesis (DPA) from cotton grown under field conditions. All of the samples were frozen in liquid nitrogen and stored at− 80 °C for RNA-Seq and gene expression analysis. We isolated each sample with at least 3–5 g from a mixture of the tissue from three individuals. Each sample includes three independent biological experiments and three technical replicates.

*N. benthamiana* seedlings were grown under 16 h light/ 8 h dark cycles, with temperatures of 25 °C during the day and 23 °C at night, for subcellular location. For the tissue location, *Arabidopsis* (Columbia) seedlings were grown in MS (Murashige and Skoog) medium at 20 °C for 7 days and subsequently transferred to nutrient soil.

### RNA sequencing and analysis

Total RNA was extracted from harvested cotton tissues using the RNAprep Pure Plant Kit (TIANGEN, Beijing, China), and examined by a NanoDrop-Photometer® spectrophotometer RNA. For the next RNA-sequencing, a total of 5 μg RNA was sent to Biomarker company (Beijing). Each sample was analyzed with three biological replicates. The sequencing protocol and analysis were performed according to the reference instructions, and were modified slightly [63]. Simply, the enriched RNA was broken into 200 nt RNA short fragments., after which the first and second-cDNA were synthesized. The synthesized double-stranded cDNA fragments were subjected to end-repair/dA-tail and ligation adapters. The suitable fragments were then purified by agarose gel electrophoresis and enriched by PCR amplification. Finally, the cDNA library was constructed using the Illumina HiSeq™ 2500 sequencing platform. The raw data were aligned to the standard line GA0024 genome using Tophat2 software [64]. The gene expression level was calculated using RPKM (reads per kilobase of exon model per million fragments mapped) through the Cufflinks software [65]. The differential expressed genes were defined by the following parameters: FDR < 0.05 and |log2 Ratio| ≥ 1. The gene ID and its sample information used in this study are listed in Additional file 6. The heat map was generated using the TBtools software.

### Plasmid construction and plant transformation

The up-stream 2000 bp promoter fragment of *GaCASP27* (*Ga08G0117*) was amplified from GA0149 genomic DNA with specific primers. The PCR fragment was subcloned into pMD19-simple T vectors for sequencing, and the correct promoter sequence was submitted to PlantCARE (http://bioinformatics.psb.ugent.be/webtools/plantcare/html/) for Cis-acting elements prediction [66]. The *GaCASP27* (*Ga08G0117*) promoter fragment was digested using *Sca*I/*Xba*I, inserted into the ScaI/XbaI sites of the PBI121 vector and replaced the 35S promoter. As a result, *pGaCASP27::GUS* recombinant vector was generated, and transformed into the *Agrobacterium tumefaciens* strain GV3101. The floral dipping method was used for the *Arabidopsis* transformation [67].

The GFP reporter vector *GaCASP27::GFP* was constructed according to the following: the full-length coding sequence (CDS) of *GaCASP27* (*Ga08G0117*) without termination codons, was amplified from GA0149 cDNA using the special primers. The PCR fragment was then subcloned into a pMD19-simple T vector and digested using the single enzyme SalI. The digested fragment cloned into linearized the pBinGFP438 vector was digested with the same enzyme to construct the plasmid *GaCASP27::GFP*, which was driven by the 35S promoter [68]. The recombinant plasmid *GaCASP27::GFP* was transferred into the *A. tumefaciens* strain LBA4404 by liquid nitrogen freezing and thawing.

### GUS histochemical staining assays and subcellular location

The homozygous *pGaCASP27::GUS Arabidopsis* positive plants were selected for tissue analysis. Seeds were grown on MS medium with 0.7% agar powder. After 2 weeks, the seedlings were soaked in a GUS staining solution containing 1 mM X-Gluc, 100 mM sodium phosphate (pH 7.0), 10 mM EDTA,0.5 mM potassium ferricyanide, 0.5 mM potassium ferrocyanide and 0.3% (v/v) Triton X-100. After staining at 37°Covernight, the tissues were decolorized using a series of different concentration gradients alcohol [69]. The samples were then photographed using optical microscopy with a CCD camera (Leica Microsystems, Germany). The GUS histochemical staining assays were performed with three biological replicates.

Strains of *A. tumefaciens* LBA4404, carrying the *GaCASP27::GFP* recombinant plasmid and the control *pBinGFP438*, were cultured overnight in an LB liquid medium containing 50 μg/mL kanamycin, 30 μg/mL rifampicin and 50 μg/mL streptomycin at 28 °C, 200 rpm. When the OD_600_ reached 0.8–1.0, the *A. tumefaciens* cells were centrifuged and suspended with infiltration buffer (10 mM MgCl_2_, 10 mM MES, pH 5.7, 200 μM acetosyringone). The suspension was then incubated at room temperature for 2 h. The recombinant plasmid and the control *pBinGFP438 A. tumefaciens* cells were infiltrated into 4 weeks old tobacco leaves by a 1 mL plastic syringe. The plants were cultured overnight in a dark environment and transferred to a chamber for 2 days with a 16 h/8 h photoperiod. The fluorescence signal in transfected tobacco leaves was observed using confocal laser scanning microscopy (Zeiss LSM510 META, Germany). Subcellular location was performed with three biological replicates.

### Virus-induced gene silencing of *GaCASP3* in tissues

*A. tumefaciens*-mediated VIGS was performed in the seedlings of GA0149. The 311 bp fragment of *Ga08G0117* was cloned into the XbaI/SacI sites of the pYL156 vector to generate the TRV:GaCASP27 recombinant plasmid. The TRV:GaCASP27 vector was transferred into the *A. tumefaciens* strain GV3101. Seeds of GA0149 cultivars were sterilized and sown in the sand to germination. After 3 days, the seedlings were transferred into plastic containers with a half-strength of Hoagland nutrient solution. When the seedlings cotyledon had completely spread, they were used for VIGS assays. The *A. tumefaciens* strain LBA4404 carrying TRV2 (helper vector), TRV1 (empty vector), TRV: CLA1 (positive control) and TRV: GaCASP27 plasmids were grown overnight in LB containing rifampicin (30 μg/mL) and kanamycin (50 μg/mL) at 28 °C, 200 rpm. When the OD_600_ value of the culture reached 1.3–1.5, the *A. tumefaciens c*ells were centrifuged and suspended in an isopyknic infiltration medium containing 10 mM MgCl_2_, 10 mM 2-(4-Morpholino) ethane sulfonic acid (MES) and 200 μM acetosyringone. After incubating for 3 h at 25 °C, the *A. tumefaciens* carrying TRV1, TRV: CLA1 and TRV: GaCASP3 was mixed with *A. tumefaciens* harboring TRV2 at a ratio of 1:1, respectively. The *A. tumefaciens* solutions were infiltrated into the two fully expanded cotyledons of the GA0149 plants via a needleless syringe. Untreated (CK) and empty vector (TRV: 00) transformed plants were used as experimental controls. The *Cloroplastos alterados1* (*CLA1*) gene was used as a positive control [70]. These infiltrated plants were grown in the greenhouse at 25 °C.

Approximately 2 weeks after infiltration, the true leaves of GA0149 were collected for RNA isolation and qRT-PCR analysis. cDNA was synthesized using a PrimeScript™ RT reagent kit with gDNA Eraser (TaKaRa, Japan). The quantitative real-time (qRT)-PCR was performed using SYBR Premix Ex Taq (TaKaRa, Japan) in a 7500 Fast Real-Time PCR system (Applied Biosystems, Inc., California USA). The *Histon3* gene was used as a reference. The relative expression levels of the *GaCASP27* gene were calculated using the 2^-ΔΔCt^ method [71]. The experiment of virus-induced gene silencing of *GaCASP27* in cotton was performed with at least three replicates.

### Assay of biomass

For assessing the biomass, 2 weeks after infiltration, the fresh seedlings of CK, TRV: 00, TRV: CLA1 and TRV: GaCASP3 were weighed using the analytical balance at the laboratory bench at room temperature. For measuring dry weight, the enzymes of the fresh seedlings were deactivated in an oven at 100–105 °C for 10 min, and then dried to a constant weight at 70 °C, after which the seedling dry weight was obtained using an analytical balance. These experiments were conducted with three biological replicates and three technical repeats.

### Analysis of root and hypocotyl traits

The lengths of the primary roots and the hypocotyls were measured using a ruler. The number of root hairs of the 15-day-old seedlings were counted using an electron microscope, and the number of lateral roots of the 15-day-old seedlings was counted using a hand magnifier. The phenotype of each root was captured using a Canon color CCD camera (EOS M5), while the color of the root was photographed using an Olympus CKX53 microscope fitted with an attached CCD camera imaging system. At least 10 lines of CK, TRV: 00, TRV: CLA1 and TRV: GaCASP3 plants were recorded for each repeat.

### Yeast one-hybrid assay

The yeast one-hybrid assay was performed according to the Matchmaker One-Hybrid System User Manual (Clontech). The 3xMYB binding elements of *GaCASP27*, attached with HindIII and SalI, were synthesized according to the primer synthesis method. The forward primers and reverse primers were mixed in a ratio of 1:1 to form a double-stranded DNA. The double-stranded DNA was cloned into the HindIII/SalI sites of pAbAi vector to generate the bait recombinant plasmid. The full length of *GaMYB36* was amplified from GA0149 cDNA using the special primers, and was then inserted into pGADT7 (Clontech) to create the prey plasmid*.* The bait plasmid was transferred into Y1HGold strains to obtain yeast strains with bait vector, while the prey plasmid was transferred into Y1HGold which contained bait vector according to the LiAc transformation method [72]. The interactions were selected on SD/−Leu plates containing 100 ng/ml Aureobasidin A (AbA), which were growth in the chamber for 3–5 days at 30 °C.

All of the gene-specific primers of the amplifications or vector constructions, as well as qRT-PCR analysis, are listed in Additional file 1.

### Statistical analysis

All of the experiments were performed with at least three replicates. Data were subjected to statistical analysis using one-way ANOVA, while Duncan’s multiple range test was used to assess the means ± SD.

## Supplementary information

**Additional file 1: Table S1.** All primers used in this study.

**Additional file 2: Table S2.** The information of *CASPs* gene family in cotton.

**Additional file 3: Table S3.** The general information of the *GaCASP* family.

**Additional file 4: Table S4.** The duplication *CASP* gene pairs in cotton.

**Additional file 5: Table S5.** Orthologous relationships between *G. arboreum* and other three *Gossypium* species.

**Additional file 6: Table S6.** The transcript expression profiles of the *GaCASP* family in GA0146 and GA0149.

**Additional file 7: Table S7.** Tissue specificity regulatory elements found in the *GaCASP27* promoter region.

**Additional file 8: Figure S1.** Chromosomal location map analysis of *CASP* genes in *G. arboreum.*

**Additional file 9: Figure S2.** pGaGASP27::GUS positive seedlings were screened in the *Kanamycin/Cephalosporin* MS medium. The red arrow indicates positive transgenic seedlings.

**Additional file 10: Figure S3.** Phenotypes of wild type*, Atmyb36*, *Atcasp1, Atcasp2* and *Atcasp3* under normal growth condition*.* **(A)** Lateral root phenotypes of WT and mutants. The seedlings were grown in vertically solid MS medium for 6 days, and transferred into MS medium for continued 7 days. **(B)** The number of the lateral roots was counted after 15 days transferring to MS. Three biological replicates were performed. The significant difference analysis was performed using one-way ANOVA and Tukey’s HSD test (*P* < 0.05).

## Data Availability

All related data are available within the manuscript and its additional files. The RNA sequences raw data were deposited in the Biological Research Project Data (BioProject), National Center for Biotechnology Information (NCBI) under the accession numbers PRJNA622455.

## Abbreviations

CASPs: Casparian stirp strip membrane domain proteins
*A. thaliana*: *Arabidopsis thaliana*
*A. tumefaciens*: *Agrobacterium tumefaciens*
VIGS: Virus-Induced Gene-Silencing
RPKM: Reads Per Kilobase of exon model per Million fragments mapped
CDS: Coding Sequence
GFP: Green Fluorescent Protein
GSDS: Gene structure display server
WGD: Whole genome duplication
Y1H: Yeast One-Hybrid assay
