# Supplementary figures and images for: Casparian strip membrane domain proteins in Gossypium arboreum: genome-wide identification and negative regulation of lateral root growth
Source: BMC Genomics. 2020 May 4;21:340. doi: 10.1186/s12864-020-6723-9 (PMC7199351; doi:10.1186/s12864-020-6723-9)

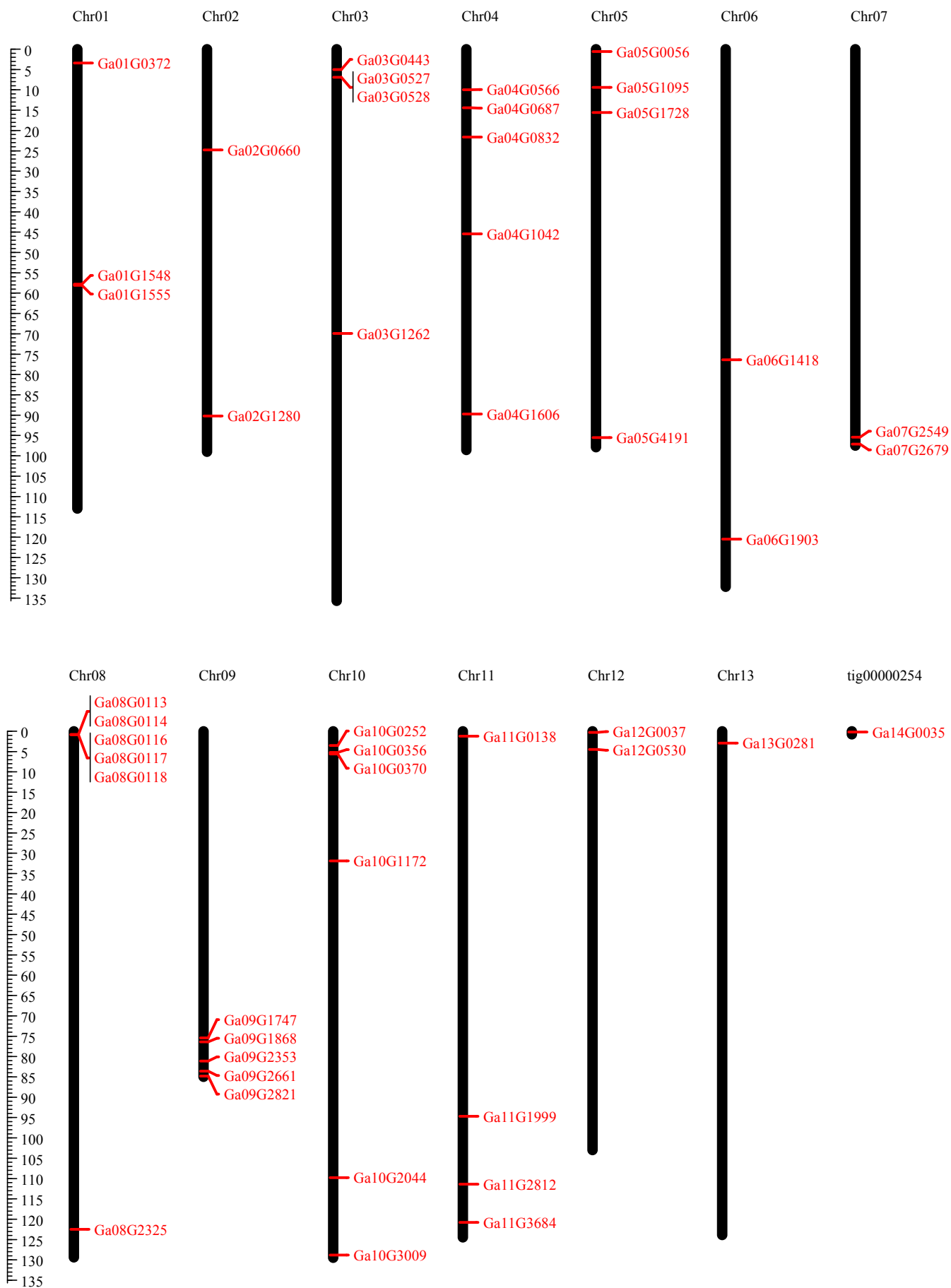

Figure S1 Chromosomal location map analysis of CASP genes in *G. arboreum*.

Supplement: Supplementary file 8 — Additional file 8: Figure S1. Chromosomal location map analysis of CASP genes in G. arboreum. [file 12864_2020_6723_MOESM8_ESM.pdf]
