# Supplementary material for: Casparian strip membrane domain proteins in Gossypium arboreum: genome-wide identification and negative regulation of lateral root growth
Source: BMC Genomics. 2020 May 4;21:340. doi: 10.1186/s12864-020-6723-9 (PMC7199351; doi:10.1186/s12864-020-6723-9)
Supplement: Supplementary file 9 — Additional file 9: Figure S2. pGaGASP27::GUS positive seedlings were screened in the Kanamycin/Cephalosporin MS medium. The red arrow indicates positive transgenic seedlings. [file 12864_2020_6723_MOESM9_ESM.pdf]

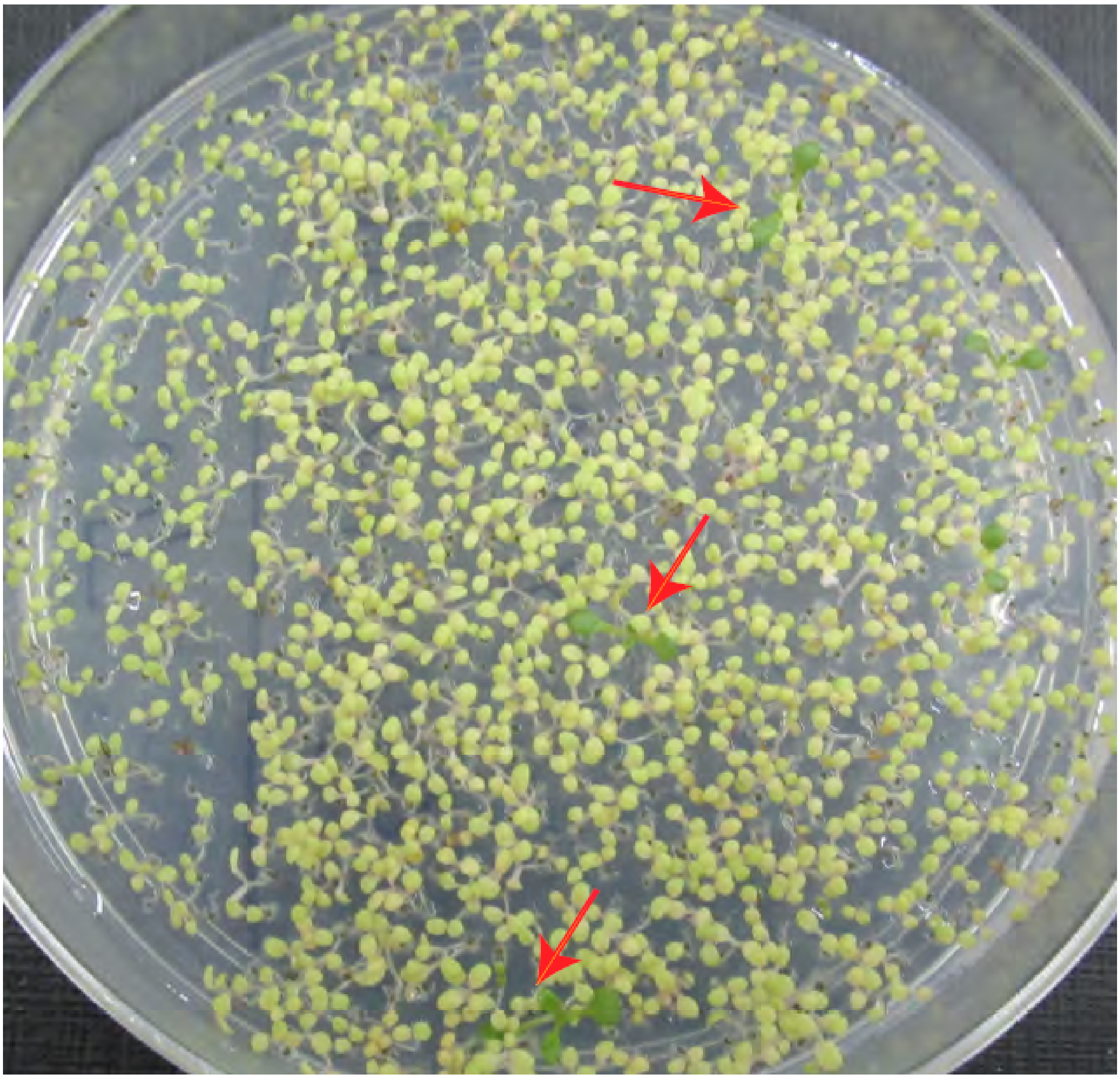

Figture S2 **pGaGASP27::GUS positive seedlings were screened in *Kanamycin/Cephalosporin* MS medium.** Red arrow indicated the positive transgenic seedlings.
