# Supplementary material for: Casparian strip membrane domain proteins in Gossypium arboreum: genome-wide identification and negative regulation of lateral root growth
Source: BMC Genomics. 2020 May 4;21:340. doi: 10.1186/s12864-020-6723-9 (PMC7199351; doi:10.1186/s12864-020-6723-9)
Supplement: Supplementary file 10 — Additional file 10: Figure S3. Phenotypes of wild type, Atmyb36, Atcasp1, Atcasp2 and Atcasp3 under normal growth condition. (A) Lateral root phenotypes of WT and mutants. The seedlings were grown in vertically solid MS medium for 6 days, and transferred into MS medium for continued 7 days. (B) The number of the lateral roots was counted after 15 days transferring to MS. Three biological replicates were performed. The significant difference analysis was performed using one-way ANOVA and Tukey’s HSD test (P < 0.05). [file 12864_2020_6723_MOESM10_ESM.pdf]

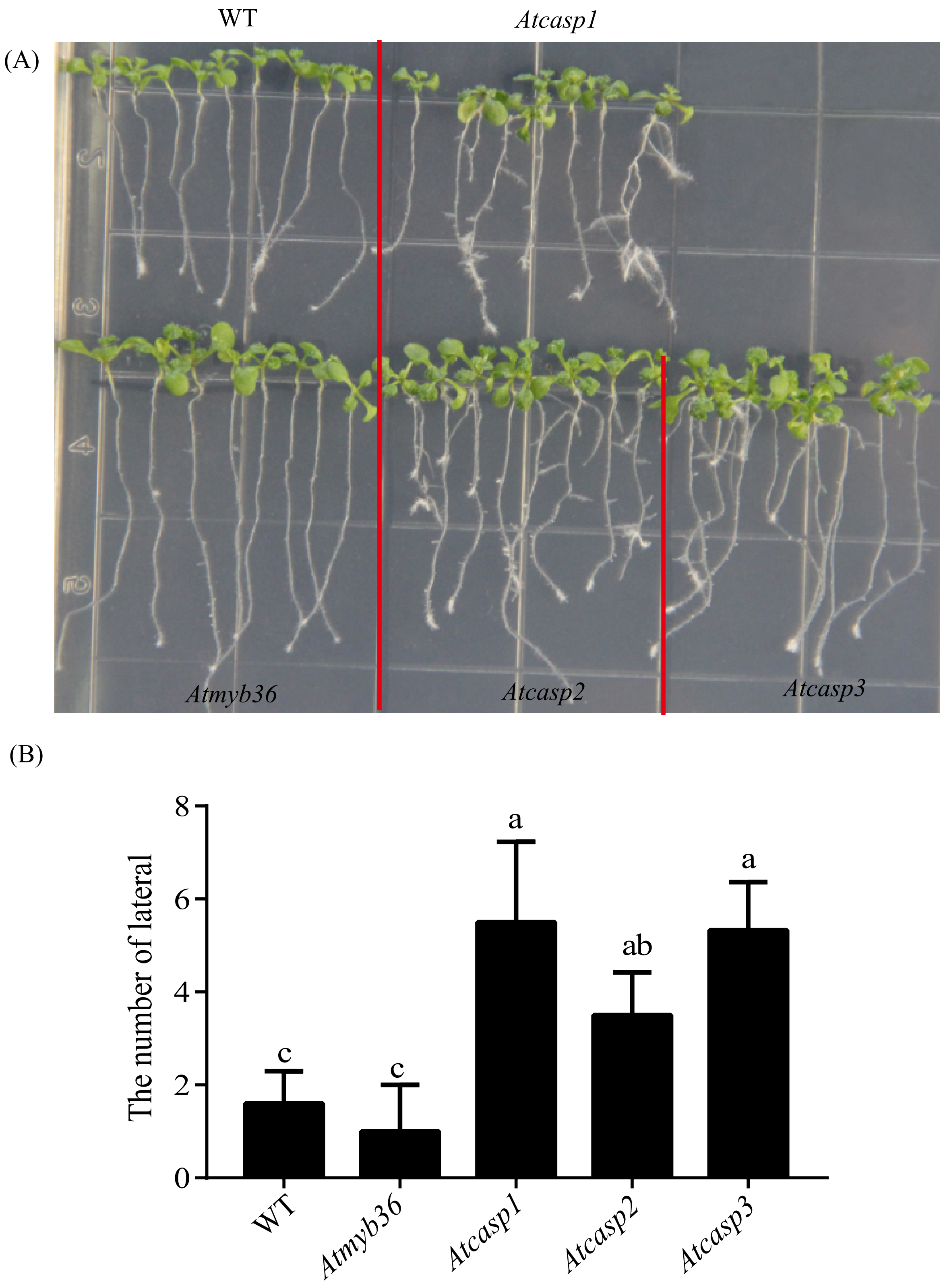

Figture S3 **Phenotypes of wild type, *Atmyb36*, *Atcasp1*, *Atcasp2* and *Atcasp3* under normal growth condition.** **(A)** Lateral root phenotypes of WT and mutants. The seedlings were grown in vertically solid MS medium for 6 days, and transferred into MS medium for continued 7 days. **(B)** The number of the lateral roots was counted after 15 days transferring to MS. Three biological replicates were performed.The significant difference analysis was performed using one-way ANOVA and Tukey’ s HSD test ($P<0.05$).
